# A randomized trial of genetic information for personalized nutrition

**DOI:** 10.1007/s12263-012-0290-x

**Published:** 2012-03-11

**Authors:** Daiva E. Nielsen, Ahmed El-Sohemy

**Affiliations:** Department of Nutritional Sciences, Room 350, University of Toronto, 150 College St, Toronto, ON M5S 3E2 Canada

**Keywords:** Nutrigenomics, Nutrigenetics, Personalized nutrition, Genetic testing

## Abstract

Personal genetic information has become increasingly accessible to the public as a result of direct-to-consumer (DTC) genetic tests; however, concerns have been raised over their value and potential risks. We compared the effects of providing genotype-based dietary advice with general recommendations on behavioral outcomes using a randomized controlled study. Participants were men and women from the Toronto Nutrigenomics and Health Study between the ages of 20–35 years (*n* = 149) who completed a survey to assess their awareness of DTC genetic tests and nutrigenomics, as well as potential motivations for undergoing genetic testing. Participants were then randomized into an intervention (I) or control (C) group and were given either genotype-based personalized dietary advice or general dietary advice, respectively. A second survey was administered to assess the participants’ opinions of the dietary reports they received. A greater proportion of participants in the intervention group agreed that they understood the dietary advice they were given (93% (I) vs. 78% (C); *p* = 0.009). Participants in the intervention group were more likely to agree that the dietary recommendations they received would be useful when considering their diet (88% (I) vs. 72% (C); *p* = 0.02) and wanted to know more about the recommendations (95% (I) vs. 76% (C); *p* < 0.0001). Only 9% of participants in the intervention group reported feeling uneasy about learning their genetic information. These findings suggest that individuals find dietary recommendations based on genetics more understandable and more useful than general dietary advice. Very few feel uneasy about receiving their genetic information that relates to personalized nutrition.

## Introduction

Recent advances in genomics technologies have made the acquisition of personalized genetic information easily obtainable. Direct-to-consumer (DTC) personal genetic tests claim to provide consumers with information about their genetic ancestry, ability to metabolize nutrients and drugs, and risk for developing diseases (Janssens and van Duijn 2010). One class of genetic tests offers personalized dietary advice based on one’s DNA to improve health (Sterling 2008). Nutrigenomics (or nutritional genomics) is the study of the relationship between genes and diet, and is used as an umbrella term for two complimentary approaches: how nutrients affect gene function and how genetic variation affects nutrient response (Cahill and El-Sohemy 2011). The latter is sometimes referred to as nutrigenetics (El-Sohemy 2007) and includes the study of how genetic variations affect food intake and eating behaviors (Eny and El-Sohemy 2010; Garcia-Bailo et al. 2009). The DTC method of marketing facilitates the sales of genetic tests without the involvement of a healthcare professional (Norrgard 2008). These tests are commercially available through the Internet and are largely unregulated, though significant measures are being taken to regulate this emerging market in certain jurisdictions (McGuire et al. 2010). The cost of the different types of genetic tests available can range from approximately $99 to over $2000 USD (Bloss et al. 2011a). DTC genetic testing for disease susceptibility remains controversial, with opponents arguing that the tests possess limited value due to their questionable clinical validity and utility (Burke 2009; Eng and Sharp 2010; Caulfield et al. 2010). Critics note that predicted risks will continue to change as new genetic variants are discovered, and thus any risk estimates for disease based on currently known common variants are premature (Janssens et al. 2011; Mihaescu et al. 2009). Moreover, environmental factors such as diet, smoking, and physical activity can have a far greater impact on risk, but are often not considered when providing estimates of risk. There is also concern that consumers may experience anxiety if provided with the estimates of higher risk for developing certain diseases based on their genes and may seek out potentially unnecessary health interventions (McGuire and Burke 2008). Another criticism of most DTC genetic tests is that the corresponding advice is not genuinely personalized since the lifestyle recommendations are generally the same, regardless of genotype. Despite these criticisms, proponents of DTC genetic tests argue that there is public interest in genomics and that individuals should have access to their own genetic information (Bloss et al. 2011a; Caulfield et al. 2010). In addition, some propose that direct access to genetic information may motivate consumers to adopt lifestyle behavioral changes aimed at reducing the risk of disease development (Bloss et al. 2011a; McBride et al. 2010). Studies have reported different findings of the effects of disclosure of genetic risk information on health-related behaviors (Arkadianos et al. 2007; Chao et al. 2008; Conradt et al. 2009; Lerman et al. 1997; Marteau et al. 2004; McBride et al. 2002; Vernarelli et al. 2010); however, only one study has investigated the impact of DTC genetic testing on behavior and reported no short-term changes in specific dietary or exercise behaviors (Bloss et al. 2011b). A limitation of that study is that the genetic risk scores that were given to the subjects were not specifically linked to a particular lifestyle behavior, and no personalized advice to reduce the risk of developing a health condition was provided. Importantly, there was no control group in the study. A recent survey of readers of the journal *Nature* shows that 27% of respondents who had their genomes analyzed changed their diet, lifestyle, or medication based on their genetic information, suggesting that genetic information could impact behavior (Maher 2011).

For appropriate recommendations and regulations regarding DTC genetic tests to be made, the public’s knowledge and opinions of these technologies need to be well understood. A number of studies have surveyed the awareness of and attitudes toward DTC genetic tests either among the general public or among healthcare providers (Cherkas et al. 2010; Goddard et al. 2009; Stewart-Knox et al. 2009; Gollust et al. 2011; Kolor et al. 2009; McGuire et al. 2009; Taylor 2011; Goddard et al. 2007). These studies report low awareness of genetic tests among the general public (13–24%) (Cherkas et al. 2010; Goddard et al. 2007, 2009), but higher awareness among healthcare providers (42–44%) (Goddard et al. 2007; Kolor et al. 2009). Studies have reported an interest in genetic testing among the public, with 50–66% of subjects reporting a willingness to undergo testing (Cherkas et al. 2010; McGuire et al. 2009; Stewart-Knox et al. 2009). Focus group research has also been conducted to better understand the knowledge and attitudes of consumers and healthcare professionals toward nutrigenomics (Morin 2009; Weir et al. 2010). Most consumers in the focus groups were unfamiliar with the term nutrigenomics and did not relate the term personalized nutrition to an individual’s genetic profile, whereas about half of healthcare professionals were aware of the term nutrigenomics (Morin 2009). After being provided with an explanation of nutrigenomics, consumers felt that a tailored diet could help reduce the risk of disease development, while healthcare professionals expressed more skepticism (Morin 2009). While these studies provide valuable insight into the public’s perceptions of nutrigenomics and genetic testing, they have all been either observational or qualitative in design. In addition, there has been some concern that genetic information obtained from a DTC genetic test is not always understood (Leighton et al. 2011), and no studies have examined whether DTC genetic tests that provide personalized nutrition advice are understandable. The objectives of the present study were to conduct a randomized controlled trial to assess behavioral outcomes as well as the awareness, perceptions, and understanding of nutrigenomics and genetic testing.

## Materials and methods

### Study design and participants

The present study is a randomized controlled trial with a 2:1 ratio of participants in the intervention versus control group. Ethics approval was obtained from the University of Toronto Institutional Review Board, and the study was registered with http://www.clinicaltrials.gov (NCT 01353014). Recruitment was carried out from May to August 2011. Participants provided informed consent by mail and then completed a baseline survey designed to assess the awareness and opinions of genetic testing and nutrigenomics using 4- and 5-point Likert scales. After the baseline survey was completed, participants were randomized to an intervention (I) or control (C) group using Random Allocation Software.

**Fig. 1:**
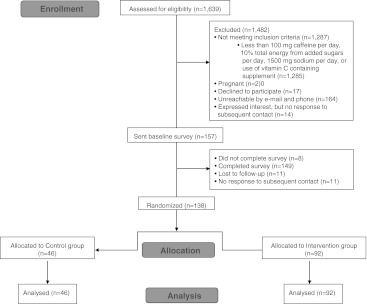
Consolidated standards of reporting trials (CONSORT) diagram of subject flow through the trial

Participants were recruited from the Toronto Nutrigenomics and Health Study (TNHS, *n* = 1,639), which is a cross-sectional study examining the role of genetics in food intake and food selection as well as gene–diet interactions on the biomarkers of chronic disease in young men and women between the ages of 20–29 years at the time of recruitment. The TNHS cohort is multi-ethnic, with participants representing three major ethnic groups: Caucasian, East Asian, and South Asian (Table 1). Recruitment for the TNHS study was carried out at the University of Toronto from 2004 to 2010. Participants provided a blood sample, and genotyping was performed for several single nucleotide polymorphisms (SNPs) involved in nutrient response and metabolism. A subset of the TNHS cohort (*n* = 354) was contacted by e-mail or phone to participate in the present study (Fig. 1). Since the recommendations in this study were based on caffeine, vitamin C, sugar, and sodium, eligible participants were those who consumed at least 100 mg of caffeine per day, 10% of total energy from added sugars per day, and 1,500 mg of sodium per day and did not take vitamin C-containing supplements. Three e-mail attempts were made, and if no response was received, one phone call was made. Eligible women who were pregnant or breast-feeding at the time of recruitment were excluded from the study.

**Table 1:** Subject characteristics

Variable	All subjects (*n* = 149)	Intervention (*n* = 92)	Control (*n* = 46)	*p*-value
	*n* (%)			
Age (years)*	26 ± 4	27 ± 3	26 ± 3	0.82
Female	113 (76)	69 (75)	37 (80)	0.48
Ethnicity				
Caucasian	92 (62)	59 (64)	24 (52)	0.18
East Asian	31 (21)	19 (21)	12 (26)	0.47
South Asian	16 (11)	9 (10)	6 (13)	0.56
Other	10 (7)	5 (5)	4 (9)	0.46
Education				
Some college or undergraduate training	20 (13)	9 (10)	8 (17)	0.20
College or undergraduate degree	76 (51)	50 (54)	22 (48)	0.47
Graduate degree	53 (36)	33 (36)	16 (35)	0.90

The *t* test statistic was used to compare the age of subjects in the intervention versus control group

The Chi-square statistic was used to compare all other characteristics of subjects in the intervention versus control group

* Values shown are mean ± standard deviation

### Intervention

Participants in the intervention group (*n* = 92) were e-mailed a personalized dietary report providing recommendations for daily intakes of caffeine, vitamin C, sugar, and sodium based on genotypes for CYP1A2 (Cornelis et al. 2006; Palatini et al. 2009), GSTM1 and GSTT1 (Cahill et al. 2009; Horska et al. 2010), TAS1R2 (Eny et al. 2010), and ACE (Poch et al. 2001), respectively. The reports were developed in collaboration with Nutrigenomix Inc. (Toronto, Canada), which is a company that is developing a nutrigenetics test kit for registered dietitians. The reports provided participants with their genotype for each gene, an explanation of what the genotype means in terms of the dietary component and a personalized recommendation for daily intake of the dietary component (Table 2). Participants in the control group (*n* = 46) received general dietary recommendations from health organizations for the same dietary components without genetic information (Table 2). After participants read the dietary report, a post-intervention survey was completed to assess their opinions of the advice they were given.

**Table 2:** Sample of dietary advice for caffeine

Intervention
Health Canada’s recommendation for caffeine is at most 300 mg/day for women of child-bearing age and at most 400 mg/day for other adults*. Since you have the CC version of the CYP1A2 gene, you might benefit from limiting your caffeine intake to no more than 200* *mg/day.* Caffeine is found in coffee, tea, cola beverages, and energy drinks. One small (8 oz) cup of coffee contains about 100 mg of caffeine, while an 8 oz cup of tea contains about 50 mg of caffeine. One can (355 ml) of cola contains about 30 mg of caffeine, while the caffeine content of energy drinks can range from 80 to 200 mg depending on the serving size and brand
Control
Health Canada’s recommendation for caffeine is at most 300 mg/day for women of child-bearing age and at most 400 mg/day for other adults. Caffeine is found in coffee, tea, cola beverages, and energy drinks. One small (8 oz) cup of coffee contains about 100 mg of caffeine, while an 8 oz cup of tea contains about 50 mg of caffeine. One can (355 ml) of cola contains about 30 mg of caffeine, while the caffeine content of energy drinks can range from 80 to 200 mg depending on the serving size and brand

### Surveys

**Fig. 2:**
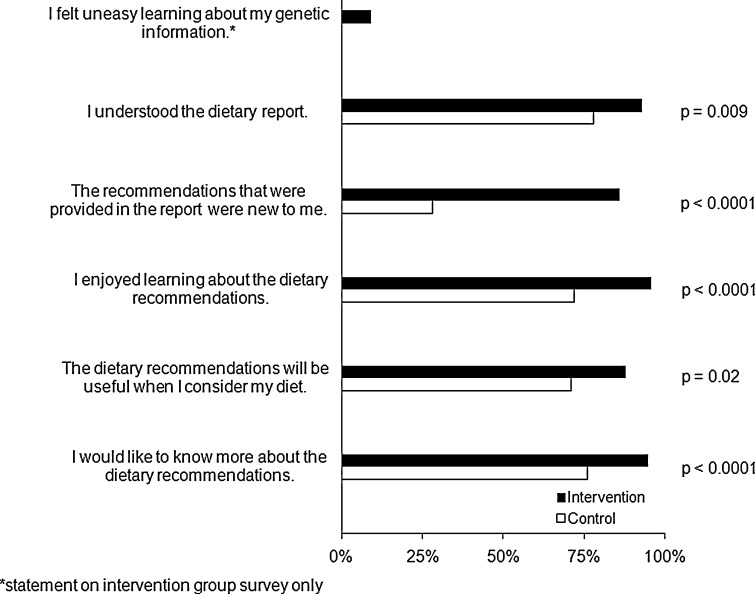
Comparison of “agree” between intervention and control group

Surveys were created using the online survey site SurveyMonkey (http://www.surveymonkey.com). Questions included were based on a literature review as well as issues raised in the Harvard University Personal Genetics Education Project (Personal genetics education project 2010). The baseline survey asked how much participants heard about DTC genetic testing to assess their awareness (Table 3). Participants were also asked how much they knew about nutrigenomics. Survey statements such as “I would take a genetic test to learn more about myself” were used to assess participants’ motivations to undergo genetic testing (Table 4). The post-intervention survey consisted of statements such as “The dietary recommendations will be useful when I consider my diet” to assess the participants’ opinions of the value of the dietary recommendations (Fig. 2).

**Table 3:** Awareness of DTC genetic tests and nutrigenomics

Question	Nothing	A little bit	A fair amount	A lot
	*n* (%)			
How much have you heard about direct-to-consumer personal genetic tests? (through media, friends, peers, etc.)	77 (52)	45 (30)	22 (15)	5 (3)
How much do you know about nutrigenomics or nutrigenetics? (the science that examines the association between genes, nutrition, and health)	44 (30)	78 (52)	22 (15)	5 (3)

**Table 4:** Attitudes toward nutrigenomics and genetic testing

Statement	Strongly agree	Somewhat agree	Neither agree nor disagree	Somewhat disagree	Strongly disagree
	*n* (%)				
I am interested in the relationship between diet and genetics	68 (46)	65 (44)	5 (3)	8 (5)	3 (2)
I would benefit from learning about how my genetic makeup affects my diet	99 (66)	32 (21)	10 (7)	4 (3)	4 (3)
Learning about my genetic makeup will affect what I eat	25 (17)	86 (58)	29 (19)	7 (5)	2 (1)
I am uncomfortable learning about my genetic makeup	11 (7)	11 (7)	13 (9)	22 (15)	92 (62)
I would take a genetic test to learn more about myself	72 (48)	56 (38)	14 (10)	5 (3)	2 (1)
I would take a genetic test to encourage myself to adopt a healthier lifestyle	67 (45)	57 (38)	13 (9)	8 (5)	4 (3)
I would take a genetic test to have my doctor monitor my health more closely	56 (38)	53 (35)	28 (19)	10 (7)	2 (1)

### Statistical analysis

Statistical analyses were performed using the Statistical Analysis Software (version 9.2; SAS Institute Inc., Cary, NC). Participants who reported “strongly agree” or “somewhat agree” to statements on the post-intervention survey were grouped (“agree”), and the Chi-square test was used to compare the frequency of “agree” to all other responses (“strongly disagree,” “somewhat disagree,” and “neither agree nor disagree”). Fisher’s exact test was used when a response category consisted of fewer than 5 counts. Significant *p* values are two-sided and less than 0.05.

## Results

### Response rate and description of participants

Of the 157 participants who were sent the baseline survey, 149 participants completed the survey giving a response rate of 95% (Fig. 1). The mean ± standard deviation age of the participants was 25 ± 4 years old and 76% were female (Table 1). The participants were highly educated with 87% having a university or college degree. Of the 149 participants who completed the baseline survey, 138 were randomized into an intervention or control group. The remaining 11 participants did not respond to subsequent e-mail attempts. There were no significant differences between the characteristics of participants in the control or intervention group (Table 1).

### Baseline survey

Approximately half of the participants (52%) reported having heard “nothing” about DTC genetic testing, while 18% reported hearing “a fair amount” or “a lot”. A smaller proportion of participants reported knowing “nothing” about nutrigenomics (30%), with just over half reporting that they knew “a little bit” about the science (52%) (Table 3). Interest in the relationship between diet and genetics was high, with 90% of participants reporting either “strongly agree” or “somewhat agree” to the survey statement. The majority of participants (87%) also agreed that they would benefit from learning about how their genetic makeup would affect their diet. Consistent with this, 75% of participants agreed that learning about their genetic makeup would affect what they ate. The greatest motivators participants reported for undergoing genetic testing were to learn more about themselves and to encourage themselves to adopt a healthier lifestyle (86 and 83%, respectively), while 73% of participants agreed that they would take a genetic test to have their doctor monitor their health more closely. Only 7% of participants strongly agreed that they would be uncomfortable learning about their genetic makeup (Table 4).

### Post-intervention survey

After receiving the dietary report, a greater proportion of participants in the intervention group agreed that they understood the dietary advice they received (93% (I) vs. 78% (C); *p* = 0.009). As expected, more participants in the intervention group agreed that the recommendations they received were new to them (86% (I) vs. 28% (C); *p* < 0.0001). Participants in the intervention group were also more likely to agree that they enjoyed learning about the recommendations (96% (I) vs. 72% (C); *p* < 0.0001), and only 9% agreed that they felt uneasy learning about their genetics (of which only one person reported “strongly agree”). In addition, participants in the intervention group were more likely to agree that the recommendations would be useful when considering their diet (88% (I) vs. 72% (C); *p* = 0.02) and that they would like to know more about the dietary recommendations they were given (95% (I) vs. 76% (C); *p* < 0.0001) (Fig. 2).

## Discussion

The results of the present study demonstrate that individuals are interested in nutrigenomics and report finding dietary recommendations based on genetics more useful than general dietary recommendations. Although concern exists over the potential for genetic information to induce anxiety in some individuals, very few participants in the intervention group agreed that they felt uneasy learning about their genetic information. Rather, 96% of participants who received their genetic information agreed that they enjoyed learning about their genetic information and dietary recommendations. This finding suggests that providing this kind of information is not likely to induce anxiety and that young adults may embrace a new era of personalized nutrition that could emerge through the advancement of personalized genomics. However, the nature of the genetic information that was provided in this study might have been perceived as less serious than genetic information related to disease risk. Although the participants in this study had an awareness of the science of nutrigenomics, only 18% reported an awareness of DTC genetic testing while 52% reported no awareness. This finding is consistent with previous surveys of the general public conducted in the UK and US (Cherkas et al. 2010; Goddard et al. 2007, 2009; Kolor et al. 2009). Despite the considerable attention DTC genetic testing has received in recent years (Lynch et al. 2011), this finding suggests that media coverage of DTC genetic testing has not yet greatly impacted young adults.

Scientific literacy and communication of genetic information are important issues to consider when studying the societal impact of DTC genetic testing (McBride et al. 2010). The literacy demands and quality of informational content across DTC genetic testing Web sites have been shown to vary (Lachance et al. 2010), and there is concern that consumers may misinterpret or not understand DTC genetic test results (Leighton et al. 2011). In the present study, a greater proportion of participants in the intervention group agreed that they understood the dietary report they were given, suggesting that dietary recommendations based on genetics can be more understandable than general dietary recommendations. This implies that providing individuals with clear, personalized nutritional advice may result in greater understanding. An important strength of the present study is the use of a randomized controlled trial, which eliminates the possibility of confounding and allows for direct comparisons to be made between experimental groups.

In considering the results of this study, some limitations should be noted. In the present study, no in-person contact was made with study participants, potentially affecting the reliability of the results. However, DTC genetic testing can be completed without in-person contact, so the nature of this study closely mimics the nature of DTC genetic testing. Seventy-six percent of participants in this study were females, and this affected our ability to report any sex-specific findings. However, excluding the males did not materially alter any of the results, suggesting that there were no major differences between men and women in this population. The age of participants in the current study was between 20 and 35 years, so findings might not be the representative of other age groups. In addition, the participants were highly educated and previously participated in a nutrigenomics study. This could explain the high degree of reported understanding of the gene-based dietary recommendations, although participants in the control group reported less understanding of the general dietary recommendations, yet were equally educated.

This study is the first to compare the impact of genotype-based personalized dietary advice with general dietary recommendations. Dietary recommendations based on genotype were reported to be more understandable than general dietary recommendations and were also reported to be more useful. Participants reported that they would not be uncomfortable learning about their own genetic information. Consistent with this, participants in the intervention group did not express discomfort in learning about their genetics and were more likely to report enjoyment in learning about the dietary recommendations they were given, as well as a greater desire to know more about the recommendations. Direct-to-consumer genetic tests based on personalized nutrition might, therefore, be more valuable that those based solely on disease risk predictions.
